# Exploring Dental Anatomy: An Ex Vivo Comparative Study Between Photon‐Counting Detector CT and Cone‐Beam CT

**DOI:** 10.1002/cre2.70266

**Published:** 2025-12-15

**Authors:** Adib Al‐Haj Husain, Fabian Benz, Victor Mergen, Silvio Valdec, Sebastian Winklhofer, Hatem Alkadhi, Harald Essig, Thomas Frauenfelder, Rubens Spin‐Neto, Bernd Stadlinger

**Affiliations:** ^1^ Department of Cranio‐Maxillofacial and Oral Surgery, University Hospital Zurich University of Zurich Zurich Switzerland; ^2^ Clinic of Cranio‐Maxillofacial and Oral Surgery, Center of Dental Medicine University of Zurich Zurich Switzerland; ^3^ Department of Cranio‐Maxillofacial Surgery, GROW School for Oncology and Reproduction Maastricht University Medical Centre Maastricht the Netherlands; ^4^ Diagnostic and Interventional Radiology University Hospital Zurich, University of Zurich Zurich Switzerland; ^5^ Department of Neuroradiology, Clinical Neuroscience Center University Hospital Zurich, University of Zurich Zurich Switzerland; ^6^ Oral Radiology, Department of Dentistry and Oral Health Aarhus University Aarhus Denmark

**Keywords:** cone‐beam computed tomography, dental anatomy, photon‐counting detector computed tomography, radiation protection

## Abstract

**Objectives:**

The aim of this ex vivo study was to assess the performance of photon‐counting detector computed tomography (PCD‐CT) compared with cone‐beam computed tomography (CBCT) at equivalent radiation doses for detecting and evaluating mandibular and dental anatomical structures in porcine cadavers.

**Material and Methods:**

This intermodal comparative study evaluated imaging protocols at three radiation dose levels (high: 360 μSv, standard: 145 μSv, low: 20 μSv) in six porcine cadaver heads, analyzing 12 CBCT and 18 PCD‐CT volumes. Two blinded observers assessed image quality, artifact susceptibility, and diagnostic interpretability using a 5‐point Likert scale (5 = highest, 1 = lowest). Statistical analysis included descriptive statistics and interobserver reliability, assessed by weighted kappa (κ) analysis.

**Results:**

PCD‐CT either matched or outperformed CBCT at standard‐ and low‐dose protocols, demonstrating superior anatomy coverage, density, contrast, and less artifact susceptibility. High‐ and standard‐dose protocols achieved perfect scores 5 (SD = 0). At low‐dose levels, PCD‐CT showed slightly lower scores but still outperformed CBCT. PCD‐CT showed minimal artifacts, with no significant artifacts in high and standard doses. Inter‐ and intra‐reader reliability was higher for PCD‐CT (κ: 0.694–1; *p* < 0.001) compared to CBCT (0.55–0.916; *p* < 0.001), with the difference being largest at low doses.

**Conclusion:**

PCD‐CT outperforms CBCT in diagnostic interpretability and artifact reduction across various radiation dose levels, offering a promising alternative for dentomaxillofacial imaging that aligns with the ALADAIP principle.

## 1 Introduction

Following a comprehensive history and detailed clinical examination, imaging has become an indispensable diagnostic tool in modern dentistry. Today, approximately 46% of all biomedical radiographs are requested in the dental field, underscoring its critical role in characterizing anatomical and diagnosing pathological conditions of the oral and maxillofacial region (Benavides et al. 2024).

In routine clinical practice, radiological workflows commonly rely on conventional single‐tooth radiographs, with radiation doses ranging between 1 and 8 μSv, or panoramic orthopantomograms (OPG), with radiation doses ranging between 4 and 30 μSv. These imaging techniques typically provide sufficient diagnostic information for most indications (Shah et al. 2014). However, managing more complex disorders often necessitates three‐dimensional information, which entails using a variety of sophisticated imaging modalities and results in higher radiation exposure. Among these modalities, cone‐beam computed tomography (CBCT) has gained widespread acceptance due to its high‐resolution, three‐dimensional visualization of mineralized structures, with radiation doses typically below 50 μSv for small to medium scanning volumes and up to 200 μSv for larger volumes (Benavides et al. 2024; Ludlow and Ivanovic 2008). Its application is particularly beneficial in endodontics, implant dentistry, and oral and maxillofacial surgery, where precise visualization and measurement of critical anatomical structures are relevant to surgical planning and prevention of complications. Thereby, CBCT is particularly effective for detecting small lesions, evaluating the cortical plate, assessing root apices and fractures, and determining the proximity of critical anatomical landmarks such as the maxillary sinus or mandibular canal—regions that conventional radiographs may not adequately capture (Weiss and Read‐Fuller 2019; Dula et al. 2014).

Despite its growing adoption, CBCT also has several limitations, including limited soft tissue contrast, uniform grayscale values, and susceptibility to artifacts from dental hardware (Kaasalainen et al. 2021; Nakamura 2020). Furthermore, although radiation doses from individual dental diagnostic procedures are relatively low, the cumulative effect of repeated biomedical imaging over a lifetime poses a significant public health concern (Hwang et al. 2018). As a result, the implementation of enhanced imaging protocols that use low radiation doses while maintaining diagnostic efficacy has gained significant momentum in recent years, guided by the “As Low As Diagnostically Acceptable being Indication‐oriented and Patient‐specific” (ALADAIP) principle (Oenning et al. 2021).

Low‐dose CBCT protocols have demonstrated potential across a range of dental applications, yielding promising results (Yeung et al. 2019). Strategies to lower radiation doses include reducing the tube current, decreasing the number of projections, or employing partial rotations. Initial feasibility studies suggest these protocols are reliable for visualizing anatomy and pathology, supporting their suitability for clinical use (Yeung et al. 2019; Kaaber et al. 2024; Pita et al. 2023). However, further research is essential to refine clinical indications, address current limitations, and understand the differences between low‐dose and conventional full‐dose protocols.

Recent advancements in computed tomography (CT) technology have led to the development of photon‐counting detectors (PCD). PCD‐CT employs cadmium telluride detectors that directly convert incident photons into electrical signals, enhancing image quality and dose efficiency (Flohr and Schmidt 2023). PCD‐CT improves the signal‐to‐noise ratio and achieves a high spatial resolution of less than 200 μm. Compared with CBCT, PCD‐CT provides high‐quality volumetric imaging with comparable spatial resolution, superior contrast for hard and soft tissues, and fewer metal‐related artifacts (Mergen et al. 2022; Al‐Haj Husain, Mergen, Sandhu et al. 2025; Sandhu et al. 2025; Al‐Haj Husain et al. 2025a). By enabling detailed visualization of small anatomical structures such as tooth roots, periodontal ligaments, and trabecular bone patterns, PCD‐CT offers significant potential for precise radiographic dental diagnosis (Vanden Broeke et al. 2021; Zanon et al. 2024; Al‐Haj Husain et al. 2025b).

The aim of this ex vivo study was to assess the performance of PCD‐CT compared with CBCT at equivalent radiation doses (low‐dose to high‐dose) for detecting and evaluating mandibular and dental anatomical structures in porcine cadavers.

## 2 Materials and Methods

### 2.1 Study Design and Ethics

Six porcine cadaver heads, sourced from a local butcher in Zurich, Switzerland, were used in this ex vivo study to provide relevant and translatable insights into the radiographic assessment of dental and mandibular anatomy. Porcine models are considered reliable in dental and orofacial research due to their physiological and anatomical similarities to the human dentomaxillofacial system (Wang et al. 2007). Therefore, they can be considered a suitable alternative for this comparative ex vivo study. The specimens were stored at −20°C and brought to room temperature prior to a series of imaging procedures performed and supervised on the same day by trained investigators (V.M., S.W., and A.A.H.).

Due to ethical considerations and radiation safety concerns, it was not feasible to perform this study in humans. While human cadavers could theoretically be used, their limited availability, strict ethical requirements, and handling restrictions make them more difficult for multiple imaging protocols in a hospital setting. Therefore, porcine cadaver heads were selected as a practical, ethically sound, and anatomically relevant model for dental imaging research. This study was conducted in accordance with the institutional guidelines of the Office of Animal Welfare and 3R of the University of Zurich for the ethical use of animal specimens in research. A formal ethical declaration of non‐responsibility was provided. This study complies with the ARRIVE (Animal Research: Reporting of in vivo Experiments) guidelines.

### 2.2 Image Acquisition

#### 2.2.1 CBCT Data Acquisition

All porcine cadaver heads were imaged using the manufacturer's predetermined standard‐dose CBCT protocol, serving as the reference standard, alongside the low‐dose CBCT imaging protocol, both performed with the Orthophos SL 3D device (Dentsply‐Sirona, Bensheim, Germany) (Sirona 2018). To ensure consistent orientation across all image volumes, each porcine mandible was centrally positioned and aligned on a platform using the scanner's positioning lights. To simulate the presence of soft tissue, a cold pack (12 × 29 cm, GELLO Geltechnik GmbH, Ahaus, Germany) was placed in the middle of each mandible. The detailed protocol parameters are presented in Table 1.

**Table 1:** Imaging protocol parameters, including the manufacturer's predefined low‐dose and standard‐dose cone‐beam computed tomography (CBCT) settings for the Orthophos SL (Dentsply Sirona, York, PA, USA) and the high‐, standard‐, and low‐dose settings for the first‐generation dual‐source photon‐counting computed tomography (PCD‐CT) system (NAEOTOM Alpha; Siemens Healthineers AG, Forchheim, Germany) used in this study.

Mode	Tube voltage (kV)	Tube current (mA)	Effective dose (μSv)
SD‐CBCT	85	13	145
LD‐CBCT	85	13	20
HD‐PCD‐CT	Sn140	104	360
SD‐PCD‐CT	Sn140	35	122
LD‐PCD‐CT	Sn140	6	20

Abbreviations: CBCT, cone‐beam computed tomography; HD, high‐dose; LD, low‐dose; PCD‐CT, photon‐counting detector computed tomography; SD, standard‐dose.

#### 2.2.2 PCD‐CT Data Acquisition

Imaging was performed using a first‐generation dual‐source photon‐counting PCD‐CT system (NAEOTOM Alpha; Siemens Healthineers AG, Forchheim, Germany), equipped with two cadmium telluride detectors. Scans were performed in the ultra‐high‐resolution mode with a detector collimation of 120 × 0.2 mm, a tube voltage of 140 kV using tin pre‐filtration, and a pitch factor of 0.85. Tube current was set to match the radiation doses of the CBCT scans. For the standard‐dose protocol, the CT volume dose index (CTDI_vol_) was adjusted to 2.4 mGy, resulting in a dose‐length product (DLP) of 61 mGy cm and an effective dose of 122 μSv, calculated using a conversion factor of 0.002 mSv mGy^−1^ cm^−1^ (Romanyukha et al. 2016). For the low‐dose protocol reduced CTDI_vol_ to 0.4 mGy, corresponding to a DLP of 10 mGy cm and an effective dose of 20 μSv. A high‐dose protocol was also included, with a CTDI_vol_ of 7.0 mGy, yielding a DLP of 180 mGy cm and an effective dose of 360 μSv (Table 1).

Scans were reconstructed as polychromatic images (T3D) with a uniform slice thickness and increment of 0.2 mm utilizing the sharp Hr76 kernel and a matrix of 1024 × 1024 pixels.

### 2.3 Image Analysis

A total of 12 CBCT volumes and 18 PCD‐CT volumes were evaluated by two observers with varying levels of experience and specialization: reader A (A.A.H.), an oral and maxillofacial surgery resident with 4 years of experience, and reader B (F.B.), a general dentist with 3 years of professional experience, performed the assessments. Prior to the evaluation, a calibration session led by one of the principal investigators was conducted to standardize the assessment process, address any potential ambiguities, and provide training on the evaluation criteria, and scoring system. All readers performed the evaluations in randomized order to ensure objective and unbiased assessments. They were blinded to each other's scoring and the imaging protocols (low‐dose, standard‐dose, and high‐dose). The assessments were repeated after a 2‐week interval to assess inter‐ and intra‐reader reliability. All evaluations were performed in a controlled environment with standardized lighting and viewing conditions, using the local Picture Archiving and Communication System (PACS) (DeepUnity Diagnost, release v.1.1.1.2, Dedalus HealthCare, Bonn, Germany).

Qualitative image assessment was performed using a modified Likert scale, as established in the literature (Sabarudin and Tiau 2013). The evaluation focused on three main aspects: anatomy coverage (5, appropriate and optimal coverage depending upon the clinical application; 4, visibility of coverage relevant to the clinical needs; 3, a sign of suspected coverage worthy for further inspection; 2, inappropriate coverage and irrelevant to clinical needs; 1, not diagnostic), density and contrast (5, excellent density and contrast between the enamel and the dentin; 4, satisfactory density and contrast between the enamel and the dentin; 3, unsatisfactory density with adequate contrast between the enamel and the dentin; 2, poor density and inadequate contrast between the enamel and the dentin; 1, not diagnostic), and the presence of artifacts (5, no artifacts; 4, minimal streaks; 3, intermediate streaks; 2, massive artifacts; 1, not diagnostic).

**Figure 1:**
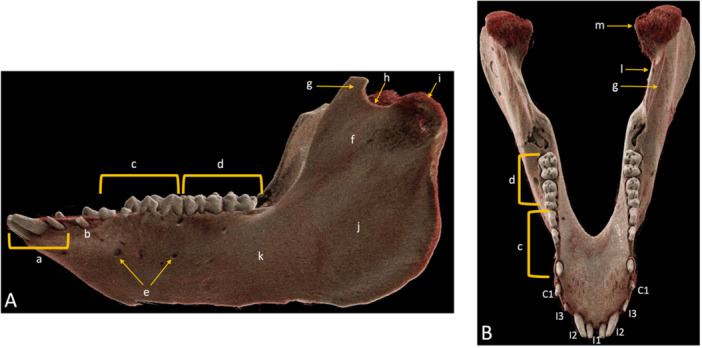
Photon‐counting detector computed tomography (PCD‐CT)‐based sagittal (A) and axial (B) cinematic rendering images of a pig cadaver, illustrating the evaluated dental and mandibular anatomy. Key structures are labeled as follows: a: incisor; b: canine; c: premolars; d: molars; e: mental foramina; f: fossa; g: coronoid process; h: mandibular notch; i: coronoid process; j: ramus; k: body; l: mandibular foramen; m: condylar process; C: canine; I: incisor.

Additionally, visualization of dental and mandibular anatomy was qualitatively assessed using a modified 5‐point Likert scale (5, fine details are visualized with full diagnostic interpretability; 4, small details are visualized with good diagnostic interpretability; 3, only broad details are visible, affecting diagnostic interpretability; 2, significant structures are not visible, allowing no diagnostic interpretability; 1, no structures are visible, with no diagnostic interpretability). The evaluation of anatomical structures adhered to established criteria in the literature (Sabarudin and Tiau 2013; Sawall et al. 2024). This encompassed the assessment of dental structures, such as enamel, dentine, the cementoenamel junction, roots, apical foramen, and the periodontal space. Additionally, mandibular structures were evaluated, including the condylar head, mandibular notch, ramus, body, angle, bony contour, spongiosa, cortical bone, mandibular foramen, mandibular canal, and mental foramen (Figure 1).

### 2.4 Statistical Analysis

Descriptive statistics were used to analyze the qualitative data related to image quality, artifacts, and visualization of dental and mandibular anatomical structures. This involved calculating means, standard deviations (SD), medians, minimums, maximums, and ranges. To assess the consistency of qualitative evaluations, both intra‐ and inter‐reader agreements were analyzed. Weighted kappa (κ) statistics were employed to measure the level of agreement beyond chance, providing a robust metric for consistency. The strength of agreement beyond chance obtained was interpreted according to the following scale: poor, < 0; slight, 0–0.2; fair, 0.21–0.4; moderate, 0.41–0.6; substantial, 0.61–0.8; almost perfect 0.81–1 (Kundel and Polansky 2003). A significance level of *α* = 0.05 was applied for all hypothesis tests. All statistical analyses were performed using IBM SPSS Statistics software (version 29.0, IBM Chicago, IL, USA).

## 3 Results

Regarding anatomy coverage, high‐dose and standard‐dose PCD‐CT achieved perfect scores (all with scores of 5), demonstrating optimal coverage across all evaluations. CBCT performed well in standard‐dose protocols with adequate coverage (4.29, SD = 0.29), but its performance decreased considerably in low‐dose protocols (3.09, SD = 0.21), showing lower values compared to low‐dose PCD‐CT (3.96, SD = 0.1).

In terms of density and contrast, PCD‐CT consistently outperformed CBCT. High‐dose and standard‐dose PCD‐CT achieved perfect scores (4.95–5), while low‐dose PCD‐CT scored lower (3.72, SD = 0.42). CBCT performed satisfactorily to excellent in the standard‐dose protocols but exhibited a more pronounced decline in low‐dose protocols compared with PCD‐CT (3.33, SD = 0.52). PCD‐CT consistently outperformed CBCT at standard and low‐dose protocols.

As for artifacts, PCD‐CT exhibited no artifacts in high‐ and standard‐dose protocols, outperforming dose‐equivalent standard‐dose CBCT protocols (5, SD = 0 vs. 4.23, SD = 0.44). In low‐dose protocols, PCD‐CT demonstrated minimal to intermediate artifacts (3.46, SD = 0.54), again surpassing low‐dose CBCT, where intermediate artifact susceptibility was more pronounced (3.08, SD = 0.21) (Table 2).

**Table 2:** Mean scores of the qualitative assessments performed by two independent readers, with both inter‐ and intra‐reader reliability. Scores were based on a modified 5‐point Likert scale. The evaluation focused on three parameters: anatomy coverage (5, appropriate and optimal for clinical application; 1, not diagnostic), density and contrast (5, excellent contrast between enamel and dentin; 1, not diagnostic), and the presence of artifacts (5, no artifacts; 1, not diagnostic). Results are presented as mean (SD) with the median in parentheses.

	Imaging protocol	Reader A1	Reader B1	Reader A2	Reader B2	Average
Anatomy coverage	SD‐CBCT	4.17 (SD = 0.41); (4)	4.33 (SD = 0.52); (4)	4.33 (SD = 0.52); (4)	4.33 (SD = 0.52); (4)	4.29 (SD = 0.49)
	LD‐CBCT	3 (SD = 0); (3)	3.17 (SD = 0.41); (3)	3.17 (SD = 0.41); (3)	3 (SD = 0); (3)	3.09 (SD = 0.21)
	HD‐PCD‐CT	5 (SD = 0); (5)	5 (SD = 0); (5)	5 (SD = 0); (5)	5 (SD = 0); (5)	5 (SD = 0)
	SD‐PCD‐CT	5 (SD = 0); (5)	5 (SD = 0); (5)	5 (SD = 0); (5)	5 (SD = 0); (5)	5 (SD = 0)
	LD‐PCD‐CT	4 (SD = 0.00); (4)	3.83 (SD = 0.41); (4)	4 (SD = 0.00); (4)	4 (SD = 0); (4)	3.96 (SD = 0.1)
Density and contrast	SD‐CBCT	4.83 (SD = 0.41); (5)	4.83 (SD = 0.41); (5)	4.83 (SD = 0.41); (5)	4.83 (SD = 0.41); (5)	4.83 (SD = 0.41)
	LD‐CBCT	3.33 (SD = 0.52); (3)	3.33 (SD = 0.52); (3)	3.33 (SD = 0.52); (3)	3.33 (SD = 0.52); (3)	3.33 (SD = 0.52)
	HD‐PCD‐CT	5 (SD = 0); (5)	5 (SD = 0); (5)	5 (SD = 0); (5)	5 (SD = 0); (5)	5 (SD = 0)
	SD‐PCD‐CT	4.83 (SD = 0.41); (5)	5 (SD = 0); (5)	5 (SD = 0); (5)	5 (SD = 0); (5)	4.95 (SD = 0.1)
	LD‐PCD‐CT	3.83 (SD = 0.41); (4)	3.5 (SD = 0.55); (3.5)	3.83 (SD = 0.41); (4)	3.7 (SD = 0.52); (4)	3.72 (SD = 0.47)
Artifacts	SD‐CBCT	4.2 (SD = 0.41); (4)	4.33 (SD = 0.52); (4)	4.2 (SD = 0.41); (4)	4.2 (SD = 0.41); (4)	4.23 (SD = 0.44)
	LD‐CBCT	3.16 (SD = 0.41); (3)	3 (SD = 0); (3)	3 (SD = 0); (3)	3.16 (SD = 0.41); (3)	3.08 (SD = 0.21)
	HD‐PCD‐CT	5 (SD = 0); (5)	5 (SD = 0); (5)	5 (SD = 0); (5)	5 (SD = 0); (5)	5 (SD = 0)
	SD‐PCD‐CT	5 (SD = 0); (5)	5 (SD = 0); (5)	5 (SD = 0); (5)	5 (SD = 0); (5)	5 (SD = 0)
	LD‐PCD‐CT	3.5 (SD = 0.55); (3.5)	3.33 (SD = 0.52); (3)	3.5 (SD = 0.55); (3.5)	3.5 (SD = 0.55; (3.5)	3.46 (SD = 0.54)

Abbreviations: CBCT, cone‐beam computed tomography; HD, high‐dose; LD, low‐dose; PCD‐CT, photon‐counting detector computed tomography; SD, standard‐dose.

**Figure 2:**
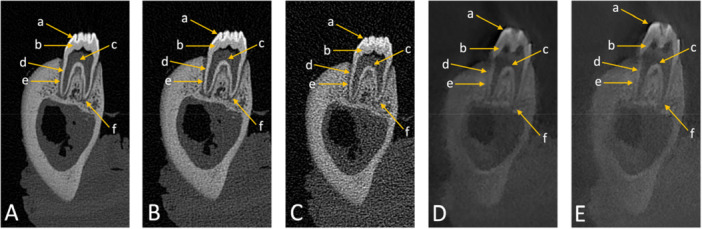
Coronal images of dental anatomy, depicting the first molar, visualized in (A) high‐dose photon‐counting detector computed tomography (PCD‐CT) reconstruction, (B) standard‐dose PCD‐CT reconstruction, and (C) low‐dose PCD‐CT reconstruction, alongside manufacturer‐specific cone‐beam computed tomography (CBCT) scans at (D) standard‐dose and (E) low‐dose protocols. Key structures are labeled as follows: a: enamel; b: dentine; c: pulp: d: root canals; e: periodontal space; f: apical foramen.

**Figure 3:**
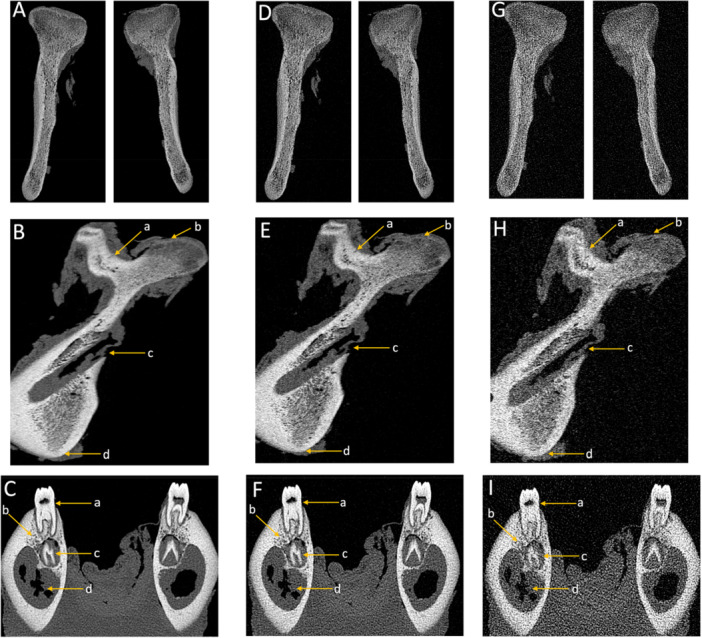
The following illustrations provide a comprehensive depiction of the mandible's intricate anatomy using photon‐counting detector computed tomography (PCD‐CT) across high‐dose (A, B, C), standard‐dose (D, E, F), and low‐dose (G, H, I) protocols. Coronal (A, D, G) and sagittal (B, E, H) views reveal the following key anatomical structures: a: mandibular notch; b: coronoid process; c: mandibular foramen; d: mandibular angle. Furthermore, coronal reconstructions (C, F, I) highlight the following structures: a: the dental complex; b: bony representation emphasizing the spongiosa and the lateral cortical bone; c: tooth follicle; d: mandibular canal.

**Figure 4:**
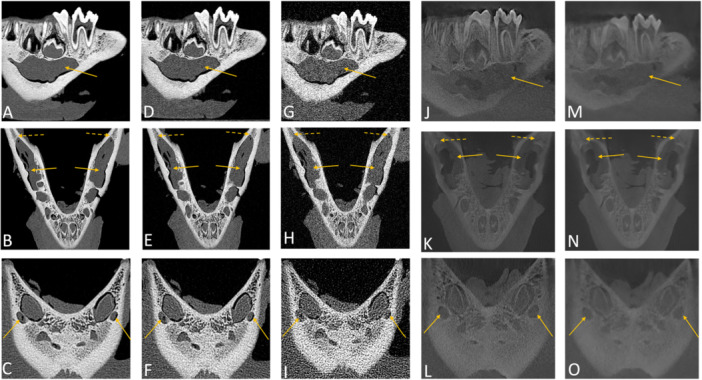
Sagittal, axial, and coronal images illustrate (A–C) high‐dose photon‐counting detector computed tomography (PCD‐CT) reconstructions, (D–F) standard‐dose PCD‐CT reconstructions, (G–I) low‐dose PCD‐CT reconstructions, and manufacturer‐specific cone‐beam computed tomography (CBCT) scans at (J–L) standard‐dose and (M–O) low‐dose protocols. The solid arrows in panels A–N indicate the location of the mandibular canal. Dotted arrows in panels B, E, H, K, and N highlight the position of the mandibular foramen. Additionally, arrows in panels C, F, I, L, and O point to the mental foramen.

**Figure 5:**
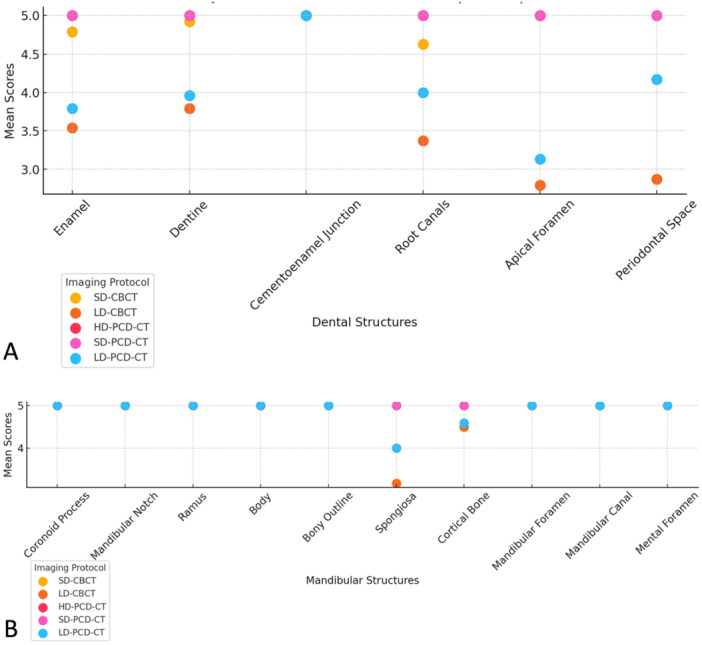
The average scores from both readers (Reader A: oral surgery resident; Reader B: general dentist) across all assessments for the qualitative evaluation of dental anatomy (A) and mandibular anatomy (B) are displayed for each imaging modality (cone‐beam computed tomography [CBCT] and photon‐counting detector ct [PCD‐CT]) along with their corresponding radiation‐equivalent imaging protocols. A modified 5‐point Likert scale was used, where a score of 5 indicates the highest diagnostic interpretability and 1 represents the least favorable interpretability.

In the assessment of dental anatomy related to diagnostic interpretability, enamel and dentin received perfect scores at both high and standard doses, while low‐dose PCD‐CT exhibited slight reductions. CBCT also showed a more pronounced decline at low dose compared with PCD‐CT. The cementoenamel junction performed optimally across all modalities, with perfect scores (5, SD = 0), showing no differences between imaging modalities or protocols. For root canals and the apical foramen, high and standard PCD‐CT provided excellent diagnostic interpretability (5, SD = 0). Low‐dose PCD‐CT scores dropped slightly (4, SD = 0 for root canals, 3.13, SD = 0.31 for apical foramen). CBCT scores were consistently lower, particularly in low‐dose protocols (3.37, SD = 0.53 for root canals, 2.79, SD = 0.44 for apical foramen). Regarding the periodontal space, high‐ and standard‐dose PCD‐CT delivered excellent results (5, SD = 0), while low‐dose PCD‐CT (4.17, SD = 0.34) and CBCT in both protocols (standard‐dose: 4.17, SD = 0.34, low‐dose: 2.87, SD = 0.31) performed less effectively (Figure 2). Diagnostic interpretability scores for mandibular anatomy were consistently optimal (5, SD = 0) across all imaging modalities and protocols for most structures, including the coronoid process, mandibular notch, ramus, body, mandibular foramen, mandibular canal, and mental foramen (Figures 3 and 4). Slight reductions were observed in low‐dose CBCT for cortical bone (4.59, SD = 0.61) and for spongiosa (3.21, SD = 0.44), while PCD‐CT maintained superior performance even in low‐dose protocols (Tables 3 and 4, Figure 5).

**Table 3:** Mean scores of the qualitative assessments of the dental anatomy, including enamel, dentin, cementoenamel junction, root canals, apical foramen, and periodontal space, were assessed by a modified 5‐point Likert scale. Scores ranged from 5, indicating the best possible value, to 1, indicating the least favorable value. The evaluation focused on diagnostic interpretability, with a score of 5 indicating that fine details are visualized with full diagnostic interpretability and a score of 1 indicating that no diagnostic interpretability is possible. Results are presented as mean (SD) with the median in parentheses.

	Imaging protocol	Reader A1	Reader B1	Reader A2	Reader B2	Average
Enamel	SD‐CBCT	4.83 (SD = 0.4); (5)	4.83 (SD = 0.41); (5)	4.67 (SD = 0.52); (5)	4.83 (SD = 0.41); (5)	4.79 (SD = 0.44)
	LD‐CBCT	3.67 (SD = 0.52); (4)	3.5 (SD = 0.55); (3.5)	3.5 (SD = 0.55); (3.5)	3.5 (SD = 0.55); (3.5)	3.54 (SD = 0.54)
	HD‐PCD‐CT	5 (SD = 0); (5)	5 (SD = 0); (5)	5 (SD = 0); (5)	5 (SD = 0); (5)	5 (SD = 0)
	SD‐PCD‐CT	5 (SD = 0); (5)	5 (SD = 0); (5)	5 (SD = 0); (5)	5 (SD = 0); (5)	5 (SD = 0)
	LD‐PCD‐CT	4 (SD = 0); (4)	3.67 (SD = 0.52); (4)	3.67 (SD = 0.52); (4)	3.83 (SD = 0.41); (4)	3.79 (SD = 0.36)
Dentine	SD‐CBCT	4.83 (SD = 0.41); (5)	5 (SD = 0); (5)	5 (SD = 0); (5)	4.83 (SD = 0.41); (5)	4.92 (SD = 0.21)
	LD‐CBCT	3.83 (SD = 0.41); (4)	3.83 (SD = 0.41); (4)	3.67 (SD = 0.52); (4)	3.83 (SD = 0.41); (4)	3.79 (SD = 0.31)
	HD‐PCD‐CT	5 (SD = 0); (5)	5 (SD = 0); (5)	5 (SD = 0); (5)	5 (SD = 0); (5)	5 (SD = 0)
	SD‐PCD‐CT	5 (SD = 0); (5)	5 (SD = 0); (5)	5 (SD = 0); (5)	5 (SD = 0); (5)	5 (SD = 0)
	LD‐PCD‐CT	4.17 (SD = 0.41); (4)	3.83 (SD = 0.41); (4)	3.83 (SD = 0.41); (4)	4 (SD = 0); (4)	3.96 (SD = 0.31)
Cementoenamel junction	SD‐CBCT	5 (SD = 0); (5)	5 (SD = 0); (5)	5 (SD = 0); (5)	5 (SD = 0); (5)	5 (SD = 0)
	LD‐CBCT	5 (SD = 0); (5)	5 (SD = 0); (5)	5 (SD = 0); (5)	5 (SD = 0); (5)	5 (SD = 0)
	HD‐PCD‐CT	5 (SD = 0); (5)	5 (SD = 0); (5)	5 (SD = 0); (5)	5 (SD = 0); (5)	5 (SD = 0)
	SD‐PCD‐CT	5 (SD = 0); (5)	5 (SD = 0); (5)	5 (SD = 0); (5)	5 (SD = 0); (5)	5 (SD = 0)
	LD‐PCD‐CT	5 (SD = 0); (5)	5 (SD = 0); (5)	5 (SD = 0); (5)	5 (SD = 0); (5)	5 (SD = 0)
Root‐canals	SD‐CBCT	4.5 (SD = 0.55); (4.5)	4.67 (SD = 0.52); (5)	4.67 (SD = 0.52); (5)	4.67 (SD = 0.52); (5)	4.63 (SD = 0.53)
	LD‐CBCT	3.33 (SD = 0.52); (3)	3.5 (SD = 0.54); (3.5)	3.33 (SD = 0.52); (3)	3.33 (SD = 0.52); (3)	3.37 (SD = 0.53)
	HD‐PCD‐CT	5 (SD = 0); (5)	5 (SD = 0); (5)	5 (SD = 0); (5)	5 (SD = 0); (5)	5 (SD = 0)
	SD‐PCD‐CT	5 (SD = 0); (5)	5 (SD = 0); (5)	5 (SD = 0); (5)	5 (SD = 0); (5)	5 (SD = 0)
	LD‐PCD‐CT	4 (SD = 0); (4)	4 (SD = 0); (4)	4 (SD = 0); (4)	4 (SD = 0); (4)	4 (SD = 0)
Apical foramen	SD‐CBCT	5 (SD = 0); (5)	5 (SD = 0); (5)	5 (SD = 0); (5)	5 (SD = 0); (5)	5 (SD = 0)
	LD‐CBCT	2.67 (SD = 0.52); (3)	2.83 (SD = 0.41); (3)	2.83 (SD = 0.41); (3)	2.83 (SD = 0.41); (3)	2.79 (SD = 0.44)
	HD‐PCD‐CT	5 (SD = 0); (5)	5 (SD = 0); (5)	5 (SD = 0); (5)	5 (SD = 0); (5)	5 (SD = 0)
	SD‐PCD‐CT	5 (SD = 0); (5)	5 (SD = 0); (5)	5 (SD = 0); (5)	5 (SD = 0); (5)	5 (SD = 0)
	LD‐PCD‐CT	3.17 (SD = 0.41); (3)	3.17 (SD = 0.41); (3)	3 (SD = 0); (3)	3.17 (SD = 0.41); (3)	3.13 (SD = 0.31)
Periodontal space	SD‐CBCT	4.17 (SD = 0.41); (4)	4 (SD = 0); (4)	4.33 (SD = 0.52); (4)	4.17 (SD = 0.41); (4)	4.17 (SD = 0.34)
	LD‐CBCT	2.83 (SD = 0.41); (3)	2.83 (SD = 0.41); (3)	3 (SD = 0); (3)	2.83 (SD = 0.41); (3)	2.87 (SD = 0.31)
	HD‐PCD‐CT	5 (SD = 0); (5)	5 (SD = 0); (5)	5 (SD = 0); (5)	5 (SD = 0); (5)	5 (SD = 0)
	SD‐PCD‐CT	5 (SD = 0); (5)	5 (SD = 0); (5)	5 (SD = 0); (5)	5 (SD = 0); (5)	5 (SD = 0)
	LD‐PCD‐CT	4 (SD = 0); (4)	4.33 (SD = 0.52)	4.17 (SD = 0.41); (4)	4.17 (SD = 0.41); (4)	4.17 (SD = 0.34)

Abbreviations: CBCT, cone‐beam computed tomography; HD, high‐dose; LD, low‐dose; PCD‐CT, photon‐counting detector computed tomography; SD, standard‐dose.

**Table 4:** Mean scores of the qualitative assessments of the diagnostic interpretability of mandibular anatomy, including the coronoid process, mandibular notch, ramus, body, bony outline/mandibular angle, spongiosa, cortical bone, mandibular foramen, mandibular canal, and mental foramen, were evaluated by the same independent readers. The assessments were based on a modified 5‐point Likert scale, with scores ranging from 5, indicating optimal diagnostic interpretability, to 1, indicating no diagnostic interpretability. Results are presented as mean (SD) with the median in parentheses.

	Imaging protocol	Reader A1	Reader B1	Reader A2	Reader B2	Average
Cornoid process (mean ± SD [median])	SD‐CBCT	5 (SD = 0); (5)	5 (SD = 0); (5)	5 (SD = 0); (5)	5 (SD = 0); (5)	5 (SD = 0)
	LD‐CBCT	5 (SD = 0); (5)	5 (SD = 0); (5)	5 (SD = 0); (5)	5 (SD = 0); (5)	5 (SD = 0)
	HD‐PCD‐CT	5 (SD = 0); (5)	5 (SD = 0); (5)	5 (SD = 0); (5)	5 (SD = 0); (5)	5 (SD = 0)
	SD‐PCD‐CT	5 (SD = 0); (5)	5 (SD = 0); (5)	5 (SD = 0); (5)	5 (SD = 0); (5)	5 (SD = 0)
	LD‐PCD‐CT	5 (SD = 0); (5)	5 (SD = 0); (5)	5 (SD = 0); (5)	5 (SD = 0); (5)	5 (SD = 0)
Mandibular notch (mean ± SD [median])	SD‐CBCT	5 (SD = 0); (5)	5 (SD = 0); (5)	5 (SD = 0); (5)	5 (SD = 0); (5)	5 (SD = 0)
	LD‐CBCT	5 (SD = 0); (5)	5 (SD = 0); (5)	5 (SD = 0); (5)	5 (SD = 0); (5)	5 (SD = 0)
	HD‐PCD‐CT	5 (SD = 0); (5)	5 (SD = 0); (5)	5 (SD = 0); (5)	5 (SD = 0); (5)	5 (SD = 0)
	SD‐PCD‐CT	5 (SD = 0); (5)	5 (SD = 0); (5)	5 (SD = 0); (5)	5 (SD = 0); (5)	5 (SD = 0)
	LD‐PCD‐CT	5 (SD = 0); (5)	5 (SD = 0); (5)	5 (SD = 0); (5)	5 (SD = 0); (5)	5 (SD = 0)
Ramus (mean ± SD [median])	SD‐CBCT	5 (SD = 0); (5)	5 (SD = 0); (5)	5 (SD = 0); (5)	5 (SD = 0); (5)	5 (SD = 0)
	LD‐CBCT	5 (SD = 0); (5)	5 (SD = 0); (5)	5 (SD = 0); (5)	5 (SD = 0); (5)	5 (SD = 0)
	HD‐PCD‐CT	5 (SD = 0); (5)	5 (SD = 0); (5)	5 (SD = 0); (5)	5 (SD = 0); (5)	5 (SD = 0)
	SD‐PCD‐CT	5 (SD = 0); (5)	5 (SD = 0); (5)	5 (SD = 0); (5)	5 (SD = 0); (5)	5 (SD = 0)
	LD‐PCD‐CT	5 (SD = 0); (5)	5 (SD = 0); (5)	5 (SD = 0); (5)	5 (SD = 0); (5)	5 (SD = 0)
Body (mean ± SD [median])	SD‐CBCT	5 (SD = 0); (5)	5 (SD = 0); (5)	5 (SD = 0); (5)	5 (SD = 0); (5)	5 (SD = 0)
	LD‐CBCT	5 (SD = 0); (5)	5 (SD = 0); (5)	5 (SD = 0); (5)	5 (SD = 0); (5)	5 (SD = 0)
	HD‐PCD‐CT	5 (SD = 0); (5)	5 (SD = 0); (5)	5 (SD = 0); (5)	5 (SD = 0); (5)	5 (SD = 0)
	SD‐PCD‐CT	5 (SD = 0); (5)	5 (SD = 0); (5)	5 (SD = 0); (5)	5 (SD = 0); (5)	5 (SD = 0)
	LD‐PCD‐CT	5 (SD = 0); (5)	5 (SD = 0); (5)	5 (SD = 0); (5)	5 (SD = 0); (5)	5 (SD = 0)
Bony outline/mandibular angle (mean ± SD [median])	SD‐CBCT	5 (SD = 0); (5)	5 (SD = 0); (5)	5 (SD = 0); (5)	5 (SD = 0); (5)	5 (SD = 0)
	LD‐CBCT	5 (SD = 0); (5)	5 (SD = 0); (5)	5 (SD = 0); (5)	5 (SD = 0); (5)	5 (SD = 0)
	HD‐PCD‐CT	5 (SD = 0); (5)	5 (SD = 0); (5)	5 (SD = 0); (5)	5 (SD = 0); (5)	5 (SD = 0)
	SD‐PCD‐CT	5 (SD = 0); (5)	5 (SD = 0); (5)	5 (SD = 0); (5)	5 (SD = 0); (5)	5 (SD = 0)
	LD‐PCD‐CT	5 (SD = 0); (5)	5 (SD = 0); (5)	5 (SD = 0); (5)	5 (SD = 0); (5)	5 (SD = 0)
Spongiosa (mean ± SD [median])	SD‐CBCT	4 (SD = 0); (4)	4 (SD = 0); (4)	4 (SD = 0); (4)	4 (SD = 0); (4)	4 (SD = 0)
	LD‐CBCT	3.17 (SD = 0.41); (3)	3.17 (SD = 0.41); (3)	3.33 (SD = 0.52); (3)	3.17 (SD = 0.41); (3)	3.21 (SD = 0.44)
	HD‐PCD‐CT	5 (SD = 0); (5)	5 (SD = 0); (5)	5 (SD = 0); (5)	5 (SD = 0); (5)	5 (SD = 0)
	SD‐PCD‐CT	5 (SD = 0); (5)	5 (SD = 0); (5)	5 (SD = 0); (5)	5 (SD = 0); (5)	5 (SD = 0)
	LD‐PCD‐CT	4 (SD = 0); (4)	4 (SD = 0); (4)	4 (SD = 0); (4)	4 (SD = 0); (4)	4 (SD = 0)
Cortical bone (mean ± SD [median])	SD‐CBCT	5 (SD = 0); (5)	5 (SD = 0); (5)	5 (SD = 0); (5)	5 (SD = 0); (5)	5 (SD = 0)
	LD‐CBCT	4.5 (SD = 0.84); (5)	4.67 (SD = 0.52); (5)	4.5 (SD = 0.55); (5)	4.67 (SD = 0.52); (5)	4.59 (SD = 0.61)
	HD‐PCD‐CT	5 (SD = 0); (5)	5 (SD = 0); (5)	5 (SD = 0); (5)	5 (SD = 0); (5)	5 (SD = 0)
	SD‐PCD‐CT	5 (SD = 0); (5)	5 (SD = 0); (5)	5 (SD = 0); (5)	5 (SD = 0); (5)	5 (SD = 0)
	LD‐PCD‐CT	5 (SD = 0); (5)	5 (SD = 0); (5)	5 (SD = 0); (5)	5 (SD = 0); (5)	5 (SD = 0)
Mandibular foramen (mean ± SD [median])	SD‐CBCT	5 (SD = 0); (5)	5 (SD = 0); (5)	5 (SD = 0); (5)	5 (SD = 0); (5)	5 (SD = 0)
	LD‐CBCT	5 (SD = 0); (5)	5 (SD = 0); (5)	5 (SD = 0); (5)	5 (SD = 0); (5)	5 (SD = 0)
	HD‐PCD‐CT	5 (SD = 0); (5)	5 (SD = 0); (5)	5 (SD = 0); (5)	5 (SD = 0); (5)	5 (SD = 0)
	SD‐PCD‐CT	5 (SD = 0); (5)	5 (SD = 0); (5)	5 (SD = 0); (5)	5 (SD = 0); (5)	5 (SD = 0)
	LD‐PCD‐CT	5 (SD = 0); (5)	5 (SD = 0); (5)	5 (SD = 0); (5)	5 (SD = 0); (5)	5 (SD = 0)
Mandibular canal (mean ± SD [median])	SD‐CBCT	5 (SD = 0); (5)	5 (SD = 0); (5)	5 (SD = 0); (5)	5 (SD = 0); (5)	5 (SD = 0)
	LD‐CBCT	5 (SD = 0); (5)	5 (SD = 0); (5)	5 (SD = 0); (5)	5 (SD = 0); (5)	5 (SD = 0)
	HD‐PCD‐CT	5 (SD = 0); (5)	5 (SD = 0); (5)	5 (SD = 0); (5)	5 (SD = 0); (5)	5 (SD = 0)
	SD‐PCD‐CT	5 (SD = 0); (5)	5 (SD = 0); (5)	5 (SD = 0); (5)	5 (SD = 0); (5)	5 (SD = 0)
	LD‐PCD‐CT	5 (SD = 0); (5)	5 (SD = 0); (5)	5 (SD = 0); (5)	5 (SD = 0); (5)	5 (SD = 0)
Mental foramen (mean ± SD [median])	SD‐CBCT	5 (SD = 0); (5)	5 (SD = 0); (5)	5 (SD = 0); (5)	5 (SD = 0); (5)	5 (SD = 0)
	LD‐CBCT	5 (SD = 0); (5)	5 (SD = 0); (5)	5 (SD = 0); (5)	5 (SD = 0); (5)	5 (SD = 0)
	HD‐PCD‐CT	5 (SD = 0); (5)	5 (SD = 0); (5)	5 (SD = 0); (5)	5 (SD = 0); (5)	5 (SD = 0)
	SD‐PCD‐CT	5 (SD = 0); (5)	5 (SD = 0); (5)	5 (SD = 0); (5)	5 (SD = 0); (5)	5 (SD = 0)
	LD‐PCD‐CT	5 (SD = 0); (5)	5 (SD = 0); (5)	5 (SD = 0); (5)	5 (SD = 0); (5)	5 (SD = 0)

Abbreviations: CBCT, cone‐beam computed tomography; HD, high‐dose; LD, low‐dose; PCD‐CT, photon‐counting detector computed tomography; SD, standard‐dose.

Inter‐ and intra‐reader agreement was consistently higher for PCD‐CT protocols compared to CBCT across all evaluated parameters. PCD‐CT demonstrated substantial to almost perfect agreement for anatomy coverage, density and contrast, and artifacts across all radiation dose protocols (average weighted kappa: 0.69–1). In contrast, CBCT showed moderate to almost perfect agreement (average weighted kappa: 0.55–0.92), with notably lower reliability observed under low‐dose conditions. For dental and mandibular anatomical structures, PCD‐CT maintained higher agreement across all dose levels, highlighting its diagnostic robustness even at reduced radiation doses (Table 5).

**Table 5:** Inter‐ and intra‐reader agreement for both readers (Reader A, an oral surgery resident; Reader B, a general dentist) regarding the qualitative parameters of anatomy coverage, density and contrast, artifacts, and the diagnostic interpretability of all dental and mandibular anatomical structures, quantified using weighted kappa values along with the corresponding 95% confidence intervals (CI).

Inter‐ and intra‐reader agreement	Imaging protocol	Reader A and B	Reader A1 and A2	Reader B1 and B2	Average
Anatomy coverage	SD‐CBCT	0.8 (0.432–1.168); *p* < 0.001	0.75 (0.596–0.924); *p* < 0.001	1 (1–1); *p* < 0.001	0.85
	LD‐CBCT	0.884 (0.671–1.097); *p* < 0.001	0.747 (0.542–0.95); *p* < 0.001	0.613 (0.457–0.868); *p* < 0.001	0.748
	HD‐PCD‐CT	1 (1–1); *p* < 0.001	1 (1–1); *p* < 0.001	1 (1–1); *p* < 0.001	1
	SD‐PCD‐CT	1 (1–1); *p* < 0.001	1 (1–1); *p* < 0.001	1 (1–1); *p* < 0.001	1
	LD‐PCD‐CT	1 (1–1); *p* < 0.001	1 (1–1); *p* < 0.001	0.788 (0.593–0.997); *p* < 0.001	0.929
Density and contrast	SD‐CBCT	0.75 (0.596–0.924); *p* < 0.001	1 (1–1); *p* < 0.001	1 (1–1); *p* < 0.001	0.916
	LD‐CBCT	0.571; (0.467–0.976); *p* < 0.001	1 (1–1); *p* < 0.001	1 (1–1); *p* < 0.001	0.857
	HD‐PCD‐CT	1 (1–1); *p* < 0.001	1 (1–1); *p* < 0.001	1 (1–1); *p* < 0.001	1
	SD‐PCD‐CT	0.784 (0.633–0.982); *p* < 0.001	0.784 (0.633–0.982); *p* < 0.001	1 (1–1); *p* < 0.001	0.856
	LD‐PCD‐CT	0.692 (0.522–0.812); *p* < 0.001	1 (1–1); *p* < 0.001	0.692 (0.522–0.812); *p* < 0.001	0.694
Artifacts	SD‐CBCT	0.647 (0.524–1.003); *p* < 0.001	1 (1–1); *p* < 0.001	0.784 (0.524–0.977); *p* < 0.001	0.81
	LD‐CBCT	0.455 (0.333–0.682); *p* < 0.001	0.625 (0.421–1.082); *p* < 0.001	0.571 (0.386–0.62); *p* < 0.001	0.55
	HD‐PCD‐CT	1 (1–1); *p* < 0.001	1 (1–1); *p* < 0.001	1 (1–1); *p* < 0.001	1
	SD‐PCD‐CT	1 (1–1); *p* < 0.001	1 (1–1); *p* < 0.001	1 (1–1); *p* < 0.001	1
	LD‐PCD‐CT	0.808 (0.508–1.065); *p* < 0.001	1 (1–1); *p* < 0.001	0.808 (0.508–1.065); *p* < 0.001	0.872
Dental anatomy	SD‐CBCT	0.751 (0.523–0.979); *p* < 0.001	0.739 (0.501–0.977); *p* < 0.001	0.809 (0.602–1.015); *p* < 0.001	0.766
	LD‐CBCT	0.624 (0.474–0.875); *p* < 0.001	0.429 (0.263–0.694); *p* < 0.001	0.517 (0.44–0.93); *p* < 0.001	0.523
	HD‐PCD‐CT	1 (1–1); *p* < 0.001	1 (1–1); *p* < 0.001	1 (1–1); *p* < 0.001	1
	SD‐PCD‐CT	1 (1–1); *p* < 0.001	1 (1–1); *p* < 0.001	1 (1–1); *p* < 0.001	1
	LD‐PCD‐CT	0.639 (0.377–0.901); *p* < 0.001	0.625 (0.354–0.895); *p* < 0.001	0.541 (0.241–0.787); *p* < 0.001	0.601
Mandibular anatomy	SD‐CBCT	0.726 (0.58–0.872); *p* < 0.001	0.8 (0.642–0.958); *p* < 0.001	0.834 (0.737–0.93); *p* < 0.001	0.787
	LD‐CBCT	0.659 (0.36–1.282); *p* < 0.001	0.659 (0.36–1.282); *p* < 0.001	0.797 (0.49–0.96); *p* < 0.001	0.705
	HD‐PCD‐CT	1 (1–1); *p* < 0.001	1 (1–1); *p* < 0.001	1 (1–1); *p* < 0.001	1
	SD‐PCD‐CT	1 (1–1); *p* < 0.001	1 (1–1); *p* < 0.001	1 (1–1); *p* < 0.001	1
	LD‐PCD‐CT	0.827 (0.694–0.961); *p* < 0.001	0.941 (0.842–1.051); *p* < 0.001	0.783 (0.493–1.072); *p* < 0.001	0.85

Abbreviations: CBCT, cone‐beam computed tomography; HD, high‐dose; LD, low‐dose; PCD‐CT, photon‐counting detector computed tomography; SD, standard‐dose.

## 4 Discussion

This ex vivo study compared the diagnostic performance of PCD‐CT with clinically established CBCT protocols at equivalent radiation dose levels for assessing mandibular and dental anatomy in pig mandibles, focusing on image quality, artifact susceptibility, and diagnostic interpretability. For all parameters, PCD‐CT demonstrated performances that were at least comparable and often superior to CBCT. This superiority was particularly evident in low‐dose imaging, where PCD‐CT outperformed radiation‐equivalent CBCT.

Studies comparing CBCT and CT in oral and maxillofacial radiology highlight each modality's indication‐specific strengths and inherent limitations, aiming to define their respective roles in clinical practice. To date, the literature on the clinical potential of PCD‐CT in intermodal comparisons is limited. As a novel imaging technology, PCD‐CT promises enhanced dose efficiency, reduced noise, and improved tissue differentiation, suggesting it could address some of the limitations inherent in CBCT imaging (Mergen et al. 2022; Sawall et al. 2024; Ruetters et al. 2022). Previous studies observed significant differences in imaging performance and noise levels between CBCT and conventional energy‐integrating detector CT. CBCT demonstrated significantly enhanced visualization of dental anatomical features, such as trabecular bone and the periodontal ligament, although with higher noise levels. No notable differences were registered when assessing larger anatomical structures, such as the mandibular canal (Liang et al. 2010; Angelopoulos et al. 2012). Additionally, CBCT demonstrated higher diagnostic accuracy than CT when using smaller fields of view (FOV), highlighting the impact of FOV size on the diagnostic performance of CBCT (Eskandarlou et al. 2014). Compared to CBCT, our study demonstrates that PCD‐CT consistently outperformed CBCT in visualizing fine dental anatomical structures, including enamel, dentin, and periodontal space. This superiority was particularly evident inlow‐dose protocols, likely due to PCD‐CT's improved contrast‐to‐noise ratio and reduced beam‐hardening artifacts (Al‐Haj Husain, Mergen, Sandhu et al. 2025; Sandhu et al. 2025; Al‐Haj Husain et al. 2025a; Sawall et al. 2024). However, no notable intermodal differences were evident in the depiction of mandibular structures, except for the imaging of the compacta and spongiosa. Thus, our findings align with studies indicating contrast‐to‐noise ratios up to 30% higher, improved sharpness in visualizing osseous structures, and enhanced qualitative ratings for PCD‐CT in illustrating dentomaxillofacial structures (Sawall et al. 2024; Klintström et al. 2024). Also, PCD‐CT's outstanding inter‐ and intra‐reader agreement and performance stand out as a valuable and reliable addition to the dental imaging diagnostic toolkit. Clinically, these advantages might make it particularly valuable for high‐precision applications, such as endodontic evaluations, implant planning, and the assessment of tooth fractures or cracks.

The results of the present study further confirm the feasibility of low‐dose protocols for diagnostic purposes in both imaging modalities. Thereby, PCD‐CT demonstrated superior image quality, contrast and density, along with reduced artifact susceptibility compared to CBCT. These findings align with existing evidence supporting the feasibility of PCD‐CT to achieve increased image quality and reduced artifact susceptibility over CBCT, even at up to a quarter of the radiation dose used in CBCT, combining minimal radiation exposure with high diagnostic accuracy [25, 31]. Furthermore, the assessed low‐dose PCD‐CT protocols might offer an innovative option for delivering standardized, detailed three‐dimensional information in dental imaging while operating at a radiation dose comparable to an OPG.

Quantitative metrics, such as contrast‐to‐noise ratio, were not included in this analysis because direct comparisons between CBCT and PCD‐CT are methodologically limited. The two modalities differ substantially in detector technology, reconstruction algorithms, voxel sizes, and gray‐value calibration, preventing standardized cross‐modal CNR assessment. In CBCT, gray values do not correspond linearly to attenuation, further reducing the interpretability of quantitative measurements. For these reasons, qualitative assessment by trained observers was deemed the most reliable approach for evaluating diagnostic performance.

This study has several limitations. Its ex vivo design was selected to address ethical and radiation safety concerns, using pig mandibles as a model. However, these specimens do not fully replicate human anatomical and physiological conditions, which may impact the generalizability of the findings. Furthermore, the lack of living tissue may influence artifact behavior and image quality, potentially limiting the translational applicability of these results to human clinical practice. Additionally, future studies incorporating intra‐modal quantitative measurements may help complement qualitative findings, particularly regarding noise behavior and the visualization of fine anatomical details. Future research should validate these findings in clinical settings, emphasizing patient‐specific imaging protocols and expanding the clinical application of low‐dose PCD‐CT protocols in dentomaxillofacial imaging.

## 5 Conclusion

This study provides evidence supporting the advantages of PCD‐CT over CBCT in dental and mandibular imaging, particularly in low‐dose protocols. PCD‐CT may pose a significant advancement in dental diagnostics, offering high‐resolution depiction and enhanced contrast‐to‐noise ratios, while simultaneously improving radiation dose efficiency. Further research aiming for clinical validation is warranted to implement PCD‐CT technology in oral and maxillofacial radiology.

## Author Contributions

Conceptualization, design, execution, and analysis: Adib Al‐Haj Husain, Fabian Benz, Victor Mergen, Silvio Valdec, Sebastian Winklhofer, Hatem Alkadhi, Harald Essig, Thomas Frauenfelder, Rubens Spin‐Neto, and Bernd Stadlinger. Drafting manuscript: Adib Al‐Haj Husain. Writing review and editing: Fabian Benz, Victor Mergen, Silvio Valdec, Sebastian Winklhofer, Hatem Alkadhi, Harald Essig, Thomas Frauenfelder, Rubens Spin‐Neto, and Bernd Stadlinger. All authors have read and agreed to the final version of the manuscript. All authors agreed to be accountable for all aspects of the work.

## Funding

The authors received no specific funding for this work.

## Disclosure

Victor Mergen, Hatem Alkadhi, and Thomas Frauenfelder received institutional research grants from Bayer, Canon, Guerbet, and Siemens. Hatem Alkadhi received speaker honoraria from Siemens.

## Ethics Statement

This study was conducted in accordance with the institutional guidelines of the Office of Animal Welfare and 3R of the University of Zurich for the ethical use of animal specimens in research. A formal ethical declaration of non‐responsibility was provided. Reporting complies with the ARRIVE guidelines.

## Consent

No human materials were used. All experiments were performed ex vivo on pig cadavers.

## Conflicts of Interest

The authors declare no conflicts of interest.

## Data Availability

The datasets used during and/or analyzed during the current study are available from the corresponding author on request.
